# Nonlinear responses of soil respiration to precipitation changes in a semiarid temperate steppe

**DOI:** 10.1038/srep45782

**Published:** 2017-03-31

**Authors:** Yuan Miao, Hongyan Han, Yue Du, Qian Zhang, Lin Jiang, Dafeng Hui, Shiqiang Wan

**Affiliations:** 1 International Joint Research Laboratory for Global Change Ecology, School of Life Sciences, Henan University, Kaifeng, Henan 475004, China; 2 School of Life Sciences, University of Chinese Academy of Sciences, Beijing 100049, China; 3 School of Biology, Georgia Institute of Technology, Atlanta, GA 30332, USA; 4 Department of Biological Sciences, Tennessee State University, Nashville, TN 37209, USA

## Abstract

Extreme precipitation events are predicted to occur more frequently and will have significant influences on terrestrial ecosystem carbon (C) cycling in the future. However, response patterns of soil respiration to precipitation changes remain uncertain in terrestrial ecosystems. A field experiment with seven precipitation treatments (i.e. from −60% to +60% of ambient precipitation to form a drought to wet precipitation gradient) was conducted over three growing seasons (2010–2012) in a semiarid temperate steppe of Northern China. Results showed a nonlinear response pattern of soil respiration along the experimental precipitation gradient, with soil respiration suppressed by decreased precipitation and enhanced by increased precipitation. Over the three growing seasons, soil respiration was reduced more under the three drought treatments (by 45.8, 32.8, and 15.9% under the −60, −40, and −20% treatments, respectively) than stimulated under the three wet treatments (by 8.9, 14.3, and 18.5% under the +20, +40, and +60% treatments, respectively). Our results indicate that soil respiration was more sensitive to decreased than increased precipitation treatments. The nonlinear and asymmetric responses of soil respiration to precipitation changes should be built into ecosystem models to project ecosystem C cycling associated with climate change.

Soil respiration is the second largest carbon (C) flux between terrestrial biosphere and the atmosphere^1^,^2^, and plays an important role in regulating soil C pools and cycling in terrestrial ecosystems^3^,^4^,^5^. Precipitation regimes are predicted to shift with increasing frequency of extreme rainfall events^6^,^7^,^8^. Previous studies have found that changing precipitation may have substantial impacts on soil respiration, as soil respiration varied greatly along precipitation gradient^9^. Given its high sensitivity to varying water availability^10^, the responses of soil respiration to precipitation changes may have substantial impacts on terrestrial ecosystem C cycling and its feedback to climate change^11^,^12^.

In arid and semiarid regions, water is the predominant limiting factor for plant growth, net primary productivity, and other biological processes^13^,^14^,^15^,^16^,^17^. Changes in precipitation may have a greater effect on soil respiration directly by impacting soil moisture and soil microbial activity, or indirectly via affecting plant growth^18^,^19^,^20^, litter decomposition^21^, and C substrate availability^22^. Irrespective of the widely reported soil respiration-precipitation observations^16^,^20^,^23^, no consensus on the relationship of soil respiration with precipitation has been reached so far. For example, increased precipitation enhances soil respiration in semiarid grasslands^12^,^24^ and in a Mediterranean shrubland^25^, but has no effects in a Northern Great Plains grassland^22^ and in an old-field grassland^26^. The different responses of soil respiration to precipitation changes could be caused by background precipitation amounts among experimental sites and precipitation treatment levels in the studies. Thus studies along a large precipitation/water availability gradient will help reveal general patterns of soil respiration-precipitation change.

Large spatial-scale investigations have demonstrated that soil respiration tends to linearly increase with precipitation amount among different sites^9^. However, model simulations showed that terrestrial ecosystems often respond nonlinearly to the driving factors of climate change including precipitation^27^,^28^,^29^. For instance, Zhou *et al*. (2008) simulated soil heterotrophic respiration and found that non-linear relationship between soil respiration and precipitation, largely due to non-proportional increases in surface runoff with increasing precipitation^30^. A meta-analysis also revealed that ecosystem C fluxes respond stronger to increased than decreased precipitation across forests, grasslands, and shrublands^31^. Nevertheless, no direct field experiments have been conducted to test soil respiration responses along a precipitation gradient in semiarid grasslands.

In this study, a field manipulative experiment was conducted over three growing seasons (June-September) of 2010–2012 to examine soil respiration responses along an experimental precipitation gradient (i.e. ambient precipitation as a control, and ±20%, ±40%, and ±60% of ambient precipitation as three drought and wet treatments) in a semiarid temperate steppe of northern China. The study site is water limited and sensitive to climate change^24^,^32^,^33^. We hypothesized that changes in precipitation/water availability could have substantial impacts on soil respiration in this ecosystem. The specific questions we tried to address: (1) Did soil respiration respond linearly or nonlinearly to changes in precipitation amount? (2) What were the underlying mechanisms influencing soil respiration response to changing precipitation?

## Results

### Variations of and effects of precipitation on soil temperature, soil moisture, soil respiration, and plant community properties

**Figure 1:**
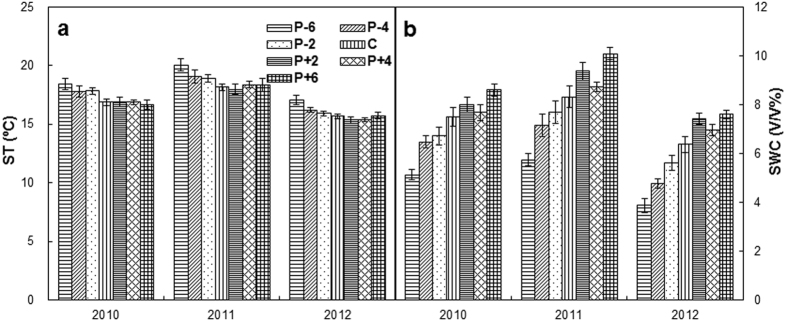
Annual variations (means ± SE) of soil temperature (ST, a) and soil moisture (SWC, b) at the depth of 10 cm in the three growing seasons, respectively. “P − 6”, “P − 4”, and “P − 2”: 60, 40, and 20% reductions in precipitation, respectively; “C”: ambient precipitation; “P + 2”, “P + 4”, and “P + 6”: 20, 40, and 60% increases in precipitation, respectively.

Soil temperature at the depth of 10 cm in the control plot was 16.9, 18.2 and 15.7 °C from 2010 to 2012 (Fig. 1), respectively. Precipitation change had no directly effects on ST but indirectly affected ST through altering the SWC (Table 1; *P* = 0.06). In contrast, precipitation changes significantly influenced SWC (Table 1). Strong interannual variations in SWC were detected (*P* < 0.001; Table 1). Over the 3 years, mean SWC were 4.92, 6.13 and 8.76% (absolute values) under the P − 6, P − 4 and P + 6 treatments. These values accounted for a decrease of 33% (*P* < 0.001) and 17% (*P* < 0.05) in the P − 6 and P − 4 treatments, respectively, and an increase of 19% (*P* < 0.01) in the P + 6 treatment, compared to that of control (Fig. 1; Table 2). There was no significant difference in SWC between the control and P − 2, P + 2 or P + 4 treatments (Table 2).

**Figure 2:**
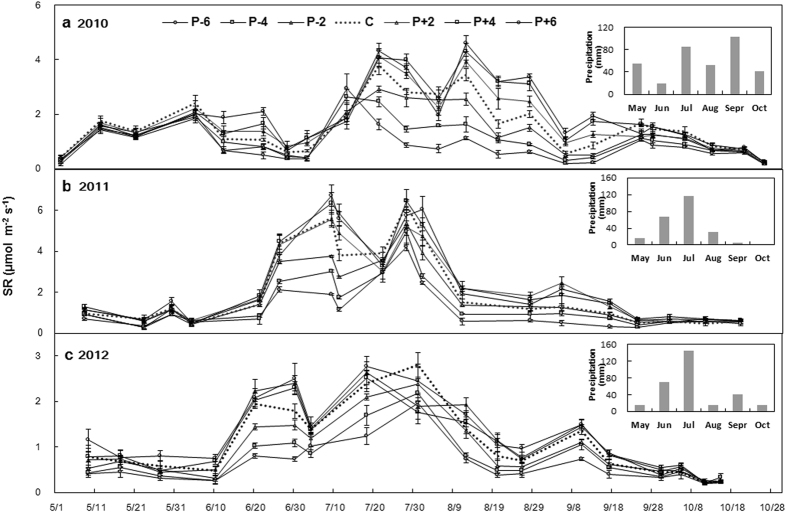
Dynamic variations of soil respiration (SR) in the three growing seasons, respectively. The inserted subplots are annual mean values (means ± 1SE). See Fig. 1 for abbreviations.

Precipitation treatments significantly affected SR (*P* < 0.001; Table 1). Mean SR were 0.89 and 1.10 μmol m^−2^ s^−1^ under the P − 6 and P − 4 treatments, respectively, which accounting for a reduction of 45.8, 32.8% (both *P* < 0.001) relative to that of control (Fig. 2; Table 2). Mean SR were 1.89 and 1.94 μmol m^−2^ s^−1^ under the P + 4 and P + 6 treatments, respectively, which accounting for an increase of 15.3 and 18.5% (both *P* < 0.05) relative to that of control (Fig. 2; Table 2). Soil respiration showed strong interannual variations (*P* < 0.001, Table 1) with the highest value (2.19 μmol m^−2^ s^−1^) appeared in 2011 and the lowest value (1.14 μmol m^−2^ s^−1^) in 2012 in the control plots (Fig. 2). Soil respiration also varied greatly during the growing seasons (from May to October), with the highest value (3.13 μmol m^−2^ s^−1^) appeared in July, and the lowest value (0.54 μmol m^−2^ s^−1^) in October in the control plots over the three growing seasons (Fig. 2). The response of SR to precipitation changes was consistent among the three growing seasons (*P* > 0.05; Table 1).

Precipitation changes also had the complex effects on community properties. Precipitation significantly affected the ANPP (*P* < 0.001) and ANPP_forb_ (*P* < 0.05). ANPP were 137.75 and 260.77 g m^−2 ^yr^−1^ under the P − 6 and P + 4 treatments, respectively, which accounting for a change of −37.28 (*P* < 0.01) and 18.37% (*P* < 0.05) relative to that of control (Table 2; Fig. S1a). No change in ANPP was detected under the P − 2, P + 2, and P + 6 treatments. ANPP_forb_ were 58.89 and 96.32 g m^−2 ^yr^−1^ under the P − 6 and P−4 treatments, respectively, which accounting for a change of −57.66 (*P* < 0.01) and −30.74% (*P* < 0.05) relative to that of control, but no differences in ANPP_forb_ were found among the other treatments (Table 2; Fig. S1b). No effects of precipitation changes on ANPP_grass_ and belowground net primary productivity (BNPP) were found (Table 3).

### Responses of soil respiration to soil water availability

**Figure 3:**
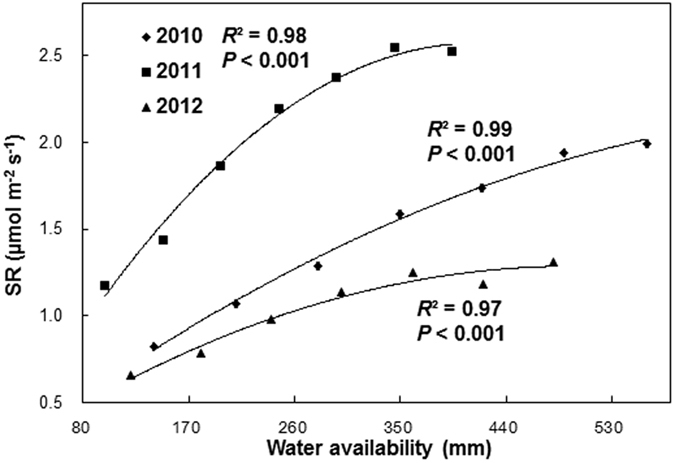
Nonlinear response patterns of soil respiration (SR) along the precipitation gradient in 2010 (y = −3E-06x^2^ + 0.005x + 0.13), 2011 (y = −1E-05x^2^ + 0.012x + 0.06), and 2012 (y = −5E-06x^2^ + 0.005x + 0.16). Each data point represents the annual mean of each treatment.

**Figure 4:**
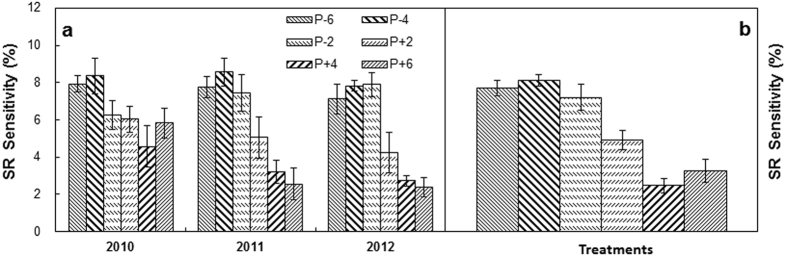
Precipitation sensitivity (percent changes per 10% precipitation change) of soil respiration (SR, annual means) in the three growing seasons, respectively (**a**) and across the three growing seasons (**b**). P−: mean sensitivity averaged across the three precipitation-reduction treatments; P+: mean sensitivity averaged across the three increased precipitation treatments; Other abbreviations see Fig. 1.

Soil respiration showed different quadratic curves along the water availability (determined by the sum of precipitation treatment and natural rainfall) in different years (Fig. 3). The sensitivity of SR to decreased precipitation was significantly higher than that to increased precipitation in each year (Fig. 4a). Over the 3 years, the mean sensitivity of SR comparing to the ambient precipitation was 7.69, 8.10 and 7.19 under the P − 6, P − 4, and P − 2 treatments, but was 4.90, 2.45 and 3.24 under the P + 2, P + 4, and P + 6 treatments, respectively. The sensitivity of SR to decreased precipitation was 2.37 times higher than that to increased precipitation over the 3 years (Fig. 4b).

### Relationships of soil respiration with abiotic and biotic factors

**Figure 5:**
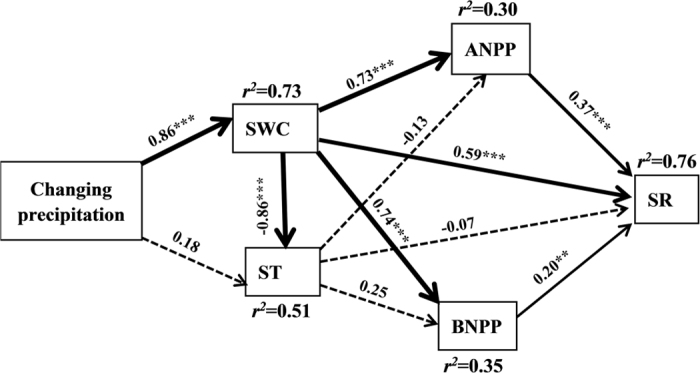
The results of structural equation model showing the causal relationships among precipitation change, soil moisture (SWC), soil temperature (ST), aboveground and belowground net primary productivity (ANPP, BNPP) to soil respiration (SR). Arrows indicate significant (solid, *P* < 0.05) and nonsignificant (dashed, *P* > 0.05) relationships. The width of arrows indicates the strength of the causal effect. Numbers above the arrows indicate path coefficients (*P < 0.05, **P < 0.01, ***P < 0.001). r^2^ values represent the proportion of variance explained for each variable. Model fit summary: χ^2^ = 2.67, P = 0.26, df = 2, GFI = 0.98, RMSEAR = 0.09.

The optimum structural equation model (SEM) revealed that the interactive networks of abiotic and biotic factors regulated SR (χ^2^ = 2.67, P = 0.26, df = 2, GFI = 0.98, RMSEAR = 0.09). Results of SEM explained 76% of the variations in SR (Fig. 5). This model also revealed that SWC not only influenced SR directly, but also affected SR indirectly by changing ANPP and BNPP. Soil temperature had no significant direct and indirect effects on SR (Fig. 5).

## Discussion

The non-linear response of soil respiration to precipitation change and the greater sensitivity of soil respiration to drought than wet treatments revealed in our study suggest that soil respiration increases to a peak with increasing precipitation, and then decreases as precipitation continues to increase in semiarid temperate steppe in China. Our findings have also showed that precipitation change not only directly impacts soil respiration by changing soil moisture, but also indirectly influences soil respiration by impacting aboveground and belowground net primary productivity. Our findings highlight that drought may have larger influences on soil respiration than precipitation increases in the semiarid temperate steppe where water availability is a primary limiting factor.

### Factors influencing soil respiration

Given that both plant and soil microbial activities are limited by water availability in semiarid grasslands^12^, precipitation-induced changes in soil respiration are closely associated with the precipitation-induced changes in soil moisture (Fig. 5). The plant root and microbial were directly influenced by soil water availability, revealing an important role of precipitation/water availability in regulating soil respiration responses in the temperate steppe. These findings are in consistent with the conclusions in several previous studies^34^,^35^,^36^. In addition to the direct effects of soil water availability on soil respiration, changes in precipitation/water availability impacted soil respiration by influencing plant growth (aboveground net primary productivity and substrate supply) and belowground C allocation (belowground net primary productivity) (Fig. 5)^14^,^35^. It is worth to note that soil temperature had no effect on soil respiration in this study (Fig. 5), which is inconsistent with a previous study that found the increased soil temperature enhances soil respiration^14^. One possible reason may be that the effect of increased soil temperature on soil respiration is context-dependency and it largely depends on initial conditions^37^. Root growths have adapted over long terms to the different soil temperature conditions, leading to the little changes of soil respiration to changes in soil temperature^38^.

### Nonlinear response of soil respiration along the precipitation gradient

The significant lower soil respiration in 2012 and 2010 than that in 2011 (Fig. 3) can be explained by the higher growing season mean soil moisture (May to October) in 2011 than that of 2010 and 2012 in the control plots, as positive dependence of soil respiration on soil moisture was founded in the control plots (Fig. S2). The non-linear response patterns of soil respiration to precipitation change in each year in our study imply that soil respiration may not monotonously increase with increasing water availability. This result is in accordance with those modeling studies that reported non-linear response patterns of heterotrophic respiration along a rainfall gradient^30^. One potential reason was that surface runoff and soil infiltration could increase exponentially with increasing precipitation amount and/or intensity^39^, causing a lower enhancement of soil respiration. However, topography at our study site is quite flat (slope <2%) with little surface runoff occurring, therefore, surface runoff might not be a factor that determining nonlinear response of soil respiration along the precipitation gradient. The sandy soil in this study may allow rainwater infiltrate easily into the deep soil. The infiltration-induced water loss could have resulted in relatively less water for plant use. In addition, increasing nitrate leaching associated with infiltration under increased precipitation might exacerbate nitrogen (N) limitation^40^, and nitrogen deficient may further limit the plant growth and soil respiration^41^. Both increasing infiltration and nitrogen deficient could lead to a nonlinear response of soil respiration to increasing precipitation. In addition, soil water availability can indirectly affect soil respiration by limiting the diffusion of soluble substrates at low water content and the diffusion of oxygen at higher water content^42^, both contributing to the nonlinear response of soil respiration along a precipitation gradient. The generalization of our results needs to be further confirmed in the future as only a few studies compared the impact of increased and decreased precipitation on soil respiration simultaneously.

### The asymmetrical sensitivity of soil respiration to the drought and wet of precipitation treatments

In our study, we found the sensitivity of soil respiration to decreased precipitation is different with that of increased precipitation. The greater sensitivity of soil respiration under the decreased than increased precipitation treatments was consistent with a study in arid and semi-arid ecosystems in North America^43^, but contradicted to the results of meta-analysis that reported higher sensitivities of ecosystem C fluxes to increased than decreased precipitation in multiple terrestrial ecosystems across different continents^20^,^31^. The possible reason for the differential soil respiration sensitivity was due to different responses of plant and soil properties in top soil, such as root biomass, available soil N, and microbial activities^10^,^44^. The synergistic effect of water and nutrients under the decreased precipitation may promote more plant growth than under increased precipitation, resulting from the contrasting amounts of available resources in root zone (i.e., primarily in shallow soil) of sandy soil. As ecosystems often experience strong variations of precipitation under natural conditions^45^, quantifying the sensitivity of ecosystem C cycling to precipitation change will improve model simulation and prediction of climate change on terrestrial C cycle.

## Conclusions

This experimental study provides a direct evidence of a nonlinear response pattern of soil respiration along an experimental precipitation gradient. Our results suggest that the rate of enhanced soil respiration by increased precipitation amounts will slow down with the higher water availability. The higher sensitivity of soil respiration under the drought condition than under the wet condition indicates that the future decline in precipitation in the temperate steppe may lead to largely changes of ecosystem C cycling. These findings not only improve the mechanistic understanding of ecosystem C cycling in response to changing precipitation regimes, but also facilitate the projections of climate change-terrestrial C cycling feedbacks in the future.

## Materials and Methods

### Experimental site

This study was carried out in a semiarid temperate steppe (42°02′N, 116°17′E, 1324 m a.s.l) in Inner Mongolia, China. The study site has been fenced to exclude grazing disturbance since 2001. Mean annual precipitation (1951–2010) was 379 mm, with 78.4% of the total precipitation fell from June to September. Mean annual temperature was 2.2 °C. The sandy soil in this area is classified as Haplic Calcisols with soil bulk density is 1.31 g cm^−3^. The vegetation type is a semiarid temperate steppe, which is composed by the dominant perennial species of *Artemisia frigida, Stipa krylovii, Potentilla acaulis, Agropyron cristatumm, and Cleistogenes squarrosa*.

### Experimental design and rainout shelters

This experiment used a randomized block design with a precipitation gradient from −60% (P − 6), −40% (P − 4), −20% (P − 2), to ambient precipitation (C), and to +20% (P + 2), +40% (P + 4), and +60% (P + 6) of ambient precipitation^46^. There were seven levels of precipitation treatments, and six replicates (blocks) for each treatment. The precipitation treatments were conducted for 4 months from June to September each year during 2010 to 2012. The precipitation treatments from −60% to +60% of ambient precipitation were selected based on the interannual fluctuation magnitudes of both annual (−34.8% to +34.8%) and June-September precipitation (−54.2% to +41.9%) over the past 60 years at the experimental site. The plot size was 4 × 4 m^2^ with a 3-m distance between any two adjacent plots, a core area with 3 × 3 m^2^ within each plot was used for measurements. V-shaped rainout shelters made by transparent plexiglass were used in the decreased precipitation plots to intercept different amounts of incoming rainfall, corresponding to different numbers of bands^47^. In the increased precipitation plots, the appropriate rainfall amount was added manually after each rainfall event. In addition, flat plexiglass bands were installed in the control and increased precipitation plots to eliminate the potential shading effects of solar radiation by plexiglass bands.

### Soil respiration, temperature, and moisture measurements

One PVC collar (11 cm in diameter and 5 cm in height) was permanently inserted at the depth of 3 cm at the center of each plot for soil respiration (SR) measurement. A LI-8100 portable soil CO_2_ flux system (Li-Cor, Inc., Lincoln, NE, USA) was used to measure SR three times per month from 09:30 AM to 12:30 PM (local time) during each year (May-October) of 2010–2012. Green plants inside the collar were cut to eliminate the effects of aboveground autotrophic respiration on the soil respiration. Soil temperature (ST) was measured with a thermocouple probe of Li-Cor 8100 when measuring SR. Volumetric soil moisture content (SWC) (0–10 cm) was measured using a portable SWC device (Diviner 2000, Sentek Pty Ltd, Balmain, Australia), recording every 3–5 days a time for a month in 2010–2012.

### Aboveground and belowground net primary productivity, plant measurements

Aboveground net primary productivity (ANPP) was estimated by clipping living biomass in August each year. All living plant tissues were harvested from two 0.15 m^2^ quadrats in each plot in August from 2010 to 2012, when plant biomass reached its peak level^44^, oven-dried at 70 °C for 48 h, and weighed. Plants were divided into two different functional groups (PFG) on the basis of growth form, including forb (ANPP_forb_) and grass (ANPP_grass_)^48^. Belowground net primary productivity (BNPP) was estimated using the root in-growth method^49^. In early May of 2010 to 2012, two 50 cm-deep cylindrical holes were excavated using a 7-cm-diameter soil augur in each plot for removing roots via 1 mm sieves, and then the soils were refilled to the same hole. Root in-growth samples were collected again in late October using a 5-cm-diameter soil augur at the center of the original root-in-growth holes. The dry mass of roots was determined by oven-drying at 70 °C until constant weight. A 1 × 1 m^2^ frame with 100 equally distributed grids (10 × 10 cm^2^) was put above the canopy in permanent quadrat (1 × 1 m^2^) in each plot for measuring plant coverage in August of 2010 to 2012. Species richness was recorded as the number of plant species in the quadrat.

### Statistical analysis

The repeated measure linear mixed-effects models were used to test the effects of year, sampling, and precipitation for ST, SWC and SR. Linear mixed-effect model was also used to test the effects of year, precipitation, for ANPP_grass_, ANPP_forb_, coverage, species richness, ANPP and BNPP. Multiple comparisons with Tukey HSD test were used for comparing the mean difference of the above variables. These statistical analyses were conducted using the *R* version 3.2.1 RC^50^. Non-linear regressions were performed to develop the relationships between SR and water availability in each year. In addition, Knapp *et al*. (2015) and Wilcox *et al*. (2015) examined the drought sensitivity of ANPP that the response of a variable (ANPP) to a unit change in a driver (millimeters of precipitation)^51^,^52^. Using a similar approach, we calculated the sensitivity of SR. The sensitivity of SR (the change of soil respiration under per 10% changes in precipitation) was calculated for each of the 6 treatments:


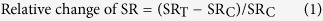



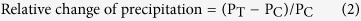



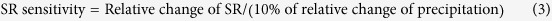


where SR_T_ is the soil respiration of precipitation treatments, SR_C_ is the soil respiration of the control; and P_T_ is the precipitation amounts of in the treatment plots, P_C_ is the precipitation amount in the ambient plots.

Furthermore, structural equation modeling (SEM) was used to identify causal linkages between explanatory variables and SR^53^. We depicted a concept model of relationship based on a prior and theoretical knowledge. All variables were ln(x + 1) transformed prior to statistical analysis for normality of data to fit the model. We then constructed SEM models that associated different variables with SR, and estimated the strength of total, direct and indirect effect of these variables (i.e. SWC, ST, ANPP and BNPP). We evaluated the fit of each model using the χ^2^-test^54^. SEM analyses were performed using AMOS 21.0 (IBM, SPSS, Armonk, NY, USA).

## Additional Information

**How to cite this article**: Miao, Y. *et al*. Nonlinear responses of soil respiration to precipitation changes in a semiarid temperate steppe. *Sci. Rep.* **7**, 45782; doi: 10.1038/srep45782 (2017).

**Publisher's note:** Springer Nature remains neutral with regard to jurisdictional claims in published maps and institutional affiliations.

## Supplementary Material

Supplementary Information

## Figures and Tables

**Table 1:** Results (*P* values) of repeated measure analysis of variance (ANOVA) using mixed-effect model on the impacts of changing precipitation and year and their interactions on soil temperature (ST), soil moisture (SWC) and soil respiration (SR), where precipitation treatment (Precipitation) is viewed as a fixed between-subjects effect, and year and sampling are viewed as repeated observations and block was viewed as a random factor.

	ST	SWC	SR
Year	0.120	**<0.001**	**<0.001**
Sampling	**<0.001**	**<0.001**	**<0.001**
Precipitation	0.060	**<0.001**	**<0.001**
Year × Sampling	**<0.001**	**<0.001**	**<0.001**
Year × Precipitation	0.899	0.571	0.224
Sampling × Precipitation	0.999	**<0.001**	**<0.001**

The *P* value that less than 0.05 are showed in bold.

**Table 2:** Results (*P* values) of multiple comparisons between changing precipitation and control (C) on soil temperature (ST), soil moisture (SWC), soil respiration (SR), aboveground net primary productivity (ANPP), ANPP of forb (ANPP_forb_) and belowground net primary productivity (BNPP) in the 3 years.

	ST	SWC	SR	ANPP	ANPP_forb_	BNPP
P − 6	0.076	**<0.001**	**<0.001**	**<0.01**	**<0.01**	0.158
P − 4	0.835	**<0.05**	**<0.001**	0.067	**<0.05**	0.757
P − 2	0.871	0.470	0.082	0.997	0.506	0.906
P + 2	0.994	0.518	0.665	0.954	0.807	0.997
P + 4	0.973	0.996	**<0.05**	**<0.05**	0.586	0.721
P + 6	0.986	**<0.01**	**<0.05**	0.944	0.104	0.592

The *P* value that less than 0.05 are showed in bold.

**Table 3:** Results (*P* values) of analysis of variance (ANOVA) using mixed-effect model for the random factor of block, fixed factors of year, precipitation, and their interactions on aboveground net primary productivity (ANPP), belowground net primary productivity (BNPP), ANPP of grass (ANPP_grass_) and forb (ANPP_forb_).

	ANPP	BNPP	ANPP_grass_	ANPP_forb_
Year	**<0.001**	0.407	**<0.001**	**<0.01**
Precipitation	**<0.001**	0.499	0.056	**<0.05**
Year × Precipitation	0.171	0.998	**<0.05**	0.638

The *P* value that less than 0.05 are showed in bold.
