# Supplementary material for: Nonlinear responses of soil respiration to precipitation changes in a semiarid temperate steppe
Source: Sci Rep. 2017 Mar 31;7:45782. doi: 10.1038/srep45782 (PMC5374450; doi:10.1038/srep45782)

**Nonlinear** **responses of soil respiration to precipitation changes** **in** **a** **semiarid temperate** **steppe**

**Yuan Miao· Hongyan Han· Yue Du· Qian Zhang· Lin Jiang· Dafeng Hui· Shiqiang Wan**

Yuan Miao· Hongyan Han· Qian Zhang· Dafeng Hui· Shiqiang Wan (🖂)

^1^International Joint Research Laboratory for Global Change Ecology, School of Life Sciences, Henan University, Kaifeng, Henan 475004, China.

E-mail: swan@henu.edu.cn

Tel: +86 371 23881006, Fax: +86 371 23882029

Yue Du

School of Life Sciences, University of Chinese Academy of Sciences, Beijing 100049, China

Lin Jiang

School of Biology, Georgia Institute of Technology, Atlanta, GA 30332, USA

Dafeng Hui

Department of Biological Sciences, Tennessee State University, Nashville, TN 37209, USA

**Supplementary Materials**

Figure S1. Annual variations (means±SE) of aboveground net primary productivity (ANPP, a), ANPP of forb (ANPP_forb_, b) and across the three growing seasons. “P-6”, “P-4”, and “P-2”: 60, 40, and 20% reductions in precipitation, respectively; “C”: ambient precipitation; “P+2”, “P+4”, and “P+6”: 20, 40, and 60% increases in precipitation, respectively.


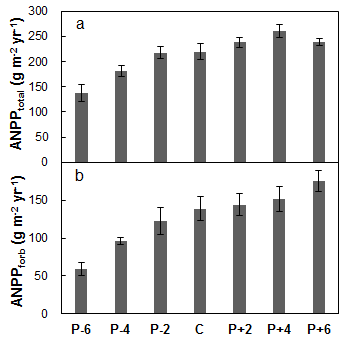


Figure S2. The relationship of SR with SWC in the control plots in three growing seasons. Each data point represents the seasonal mean value of control plot.


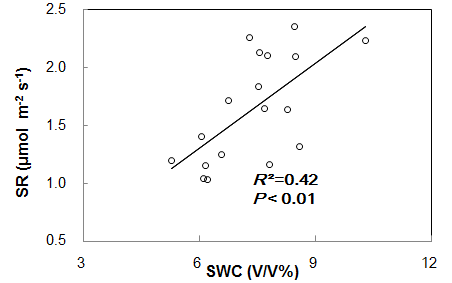

Supplement: Supplementary Information [file srep45782-s1.doc]
